# Comparative genomic and metabolomic study of three *Streptomyces* sp. differing in biological activity

**DOI:** 10.1002/mbo3.1389

**Published:** 2023-10-30

**Authors:** Alisson Gillon, Ola Abdelrahman, Eliane Abou‐Mansour, Floriane L'Haridon, Laurent Falquet, Pierre‐Marie Allard, Laure Weisskopf

**Affiliations:** ^1^ Department of Biology University of Fribourg Fribourg Switzerland; ^2^ Department of Botany University of Khartoum Khartoum Sudan; ^3^ Genes and genomes Swiss Institute of Bioinformatics Lausanne Switzerland

**Keywords:** borrelidin, genomics, metabolomics, *Phytophthora infestans*, *Streptomyces*

## Abstract

The *Streptomyces* genus is known to produce many specialized metabolites of value for medicine, but the potential of these metabolites in agronomy remains largely unexplored. In this study, we investigated three phylogenetically closely related *Streptomyces* strains (B5, B91, and B135) isolated from three distinct soil samples in Sudan. Despite belonging to the same species, these strains exhibited different ranges of *Phytophthora infestans* inhibition. The objective of this work was to identify the active compound(s) responsible for the inhibition of *P. infestans* and of other plant pathogens by comparing the genomes and metabolomes of the three strains which showed distinct activity patterns: B5 was the strongest inhibitor of oomycetes, B5 and B91 both inhibited most fungi and B135 was the only strain showing antibacterial activity. Our comparative genomic and metabolomic analysis identified borrelidin as the bioactive compound underlying B5's strong anti‐oomycete activity and highlighted a few other metabolites as putative candidates underlying the strains' antifungal and antibacterial activities. This study illustrates the power of comparative genomics and metabolomics on phylogenetically closely related strains of differing activities to highlight bioactive compounds that could contribute to new sustainable crop protection strategies.

## Graphical abstract

This work demonstrates that the analysis of the metabolomes and the genomes of phylogenetically closely related strains which show different phenotypes can lead in a straightforward way to elucidate the compounds responsible for the observed phenotypes. Using comparative metabolomics coupled to molecular networking on three *Streptomyces* strains, we identified borrelidin as the active compound underlying the inhibition of *Phytophthora infestans* and other fungal plant pathogens.

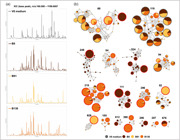

## 1 INTRODUCTION

The *Streptomyces* genus, which belongs to the Actinomycetales order within the Actinobacteria phylum, consists of spore‐forming Gram‐positive bacteria able to disseminate through wind and water, as well as to survive under extreme environmental conditions (Barka et al., 2016). Moreover, these organisms have been demonstrated to produce a broad spectrum of specialized metabolites and observed to promote plant growth as well as to help plants in their fight against pathogens (Chen et al., 2018; Law et al., 2017; Newitt et al., 2019; Rey & Dumas, 2017; Vijayabharathi et al., 2018; Vurukonda et al., 2018). This ability of *Streptomyces* strains to produce highly bioactive specialized metabolites, together with their capacity to endure harsh environments such as plant surfaces and to survive as spores, which would facilitate formulation, makes them promising candidates for agronomical application. However, compared with the extensive body of studies investigating the use of their metabolites for human medicine, their potential for application as biocontrol agents remains largely underexploited (Barka et al., 2016; van der Meij et al., 2017; Rey & Dumas, 2017). Nevertheless, a few *Streptomyces* spp.‐based biocontrol products have been successfully commercialized worldwide, such as Actinovate®, Mycostop®, Mykocide KIBC Co.®, Safegrowth KIBC Co.®, Actofit®, Bactofil®, Bialaphos®, and Incide SP®. Additionally, several bioactive compounds derived from *Streptomyces* spp. are already available in the market, including Blasticidin‐S, Kasugamycin, Streptomycin, Phytomycin, Validamycin, Polyoxorim, Natamycin, Abamectin, Polynactin, and Milbemycin (Barka et al., 2016; Palaniyandi et al., 2013; Vurukonda et al., 2018). These products, which target a wide range of organisms including fungi, oomycetes, bacteria, weeds, and insects, emphasize the potential of using either the strains themselves or their metabolites for crop protection.

One of the major phytopathogens that pose a significant threat to the Solanaceae family, particularly tomatoes and potatoes, is the oomycete *Phytophthora infestans*, responsible for the late blight disease (Fry, 2008; Hashemi et al., 2021; Kamoun et al., 2015; Tsedaley, 2014). According to FAOSTAT (2023), potatoes were grown on approximately 18.1 million hectares in 2021, highlighting the importance of protecting this crop. Hence, effective and sustainable control measures against late blight are crucial, as this disease represents a major hurdle to potato production, impacting both the economy and food security (Dong & Zhou, 2022; De Vrieze et al., 2018).

In an earlier study, a collection of 175 Actinomycetes strains isolated from different Sudanese habitats were assessed for their inhibitory activity on *P. infestans* (Abdelrahman et al., 2022). Within this collection, we observed that three *Streptomyces* strains that showed close phylogenetic relationships based on 16S ribosomal RNA (rRNA) gene homology displayed very different levels of *P. infestans* mycelial growth inhibition. However, the inhibitory activity of these three strains was not further investigated beyond *P. infestans*. The present study investigated the inhibitory potential of these three *Streptomyces* strains against a wide range of other economically relevant phytopathogens that cause severe crop diseases and belong to various divisions and phyla, including Oomycota, Basidiomycota, Ascomycota, and Bacteria. Within the Oomycota group, *Phytophthora capsici* is known to cause wilt in members of the Solanaceae family, particularly peppers and tomatoes (Kamoun et al., 2015; Trinidad‐Cruz et al., 2021), while *Pythium ultimum* induces damping off and root rot in a wide range of plant hosts such as soybean and wheat (Kamoun et al., 2015). *Rhizoctonia solani*, belonging to the Basidiomycota, is responsible for black scurf and stem canker in potato plants, as well as in various alternative hosts (Dean et al., 2012; Tsror, 2010). Among the Ascomycota, *Fusarium oxysporum* causes Fusarium wilt in diverse plant species (Dean et al., 2012; Lombard et al., 2019), *Botrytis cinerea* leads to gray mold and affects over 200 plant species (Bruisson et al., 2019; Dean et al., 2012), and *Alternaria solani* acts as the causal agent of early blight in potato plants (Chaerani & Voorrips, 2006; Parvin et al., 2021). In addition to the above‐mentioned fungi and oomycetes, the present study also examined the effect of the three selected *Streptomyces* strains on two bacterial pathogens, *Dickeya solani*, and *Pectobacterium carotovorum*, responsible for inducing blackleg disease and soft rot of potato tubers (Mansfield et al., 2012; Pédron et al., 2021; Toth et al., 2011).

Since we expected the three closely related *Streptomyces* strains to differ in their activity not only on *P. infestans* but also on other pathogens, the present work aimed to identify the active compound(s) involved in the inhibition of the various pathogens, by mining the *Streptomyces* genomes and metabolomes for biosynthetic gene clusters (BGCs) and chemical compounds specifically associated with the respective pathogen‐inhibiting activity.

## 2 EXPERIMENTAL PROCEDURES

### 2.1 Biological material and culture conditions

Three *Streptomyces* strains (B5, B91, and B135) isolated in Sudan and originating from different habitats were used in this project. Their isolation and characterization were described in Abdelrahman et al. (2022). They were routinely grown at 28°C on V8 medium (Miller, 1955) containing (per liter of distilled water) 1 g of CaCO_3_ (BioXtra ≥ 99%; Sigma‐Aldrich), 15 g of agar (Carl Roth), and 100 mL of V8 100% spicy hot vegetable juice (Campbell Soup Company). For their use in this study, spore suspensions were prepared as described earlier (Abdelrahman et al., 2022) and their optical density (OD) was adjusted to OD_600_ = 0.1 by the addition of sterile distilled water.

A majority of the pathogens used in this study were provided by Nicole Lecoultre from Agroscope (Nyon): isolate 2713 of *A. solani*, isolate 212 of *P. ultimum*, isolate Imp16/645‐4 of *P. carotovorum*, and isolate CH16/120‐3 of *D. solani*. All organisms were grown on a V8 medium. The two bacterial pathogens were incubated at room temperature for 3 days before use. *A. solani* was incubated for 2 weeks at 18°C in the dark and *P. ultimum* was incubated at room temperature for 2 days before use. The isolate Rec01 of *P. infestans* was provided by Heinz Krebs from Agroscope (Zurich‐Affoltern). This phytopathogen was incubated at 18°C for 2 weeks before use. The fungi *F. oxysporum* strain 5704 and *R. solani* strain 5900 were provided by Syngenta SA (Basel). They were incubated at 18°C in the dark for 1 week and 3 days, respectively. The BMM isolate of *B. cinerea* was provided by Prof. Brigitte Mauch‐Mani from the University of Neuchâtel. This fungus was incubated at 20°C with a 12‐h light/dark cycle alternating every 12 h for 3–5 days. Finally, the isolate Pc263 of *P. capsici* was provided by Iga Tomczynska from Brett Tyler's laboratory located at Oregon State University. It was incubated at 21°C in the dark for 5–7 days. In addition to the V8 medium, the LB medium was used for some experiments. LB medium was prepared by mixing 12.5 g of LB Broth Miller (LB‐medium [Luria‐Miller]; Carl Roth) and 10 g of LB Broth Lennox (LB‐Medium [Lennox]; Carl Roth) in 1 L of distilled water, followed by autoclaving at 121°C and 15 psi for 20 min.

### 2.2 Genomic comparison of the three *Streptomyces* strains

#### 2.2.1 Whole genome extraction

Each *Streptomyces* strain was inoculated into 20 mL tryptic soy broth medium (Sigma‐Aldrich) and incubated at 28°C under shaking (180 rpm) for 3 days. DNA was extracted using the GenElute™ Bacterial Genomic DNA Kit (Merck), according to the manufacturer's instruction with slight modifications: the cells were incubated with the lysozyme solution for 90 min instead of 30 min, they were then incubated with proteinase K and the lysis solution C for 15 min instead of 10 min. The quality and quantity of the extracted DNA samples were evaluated using a 1% agarose gel electrophoresis and a Nanodrop, respectively.

#### 2.2.2 Genome sequencing and assembly

The genomic DNA was processed by the Next‐Generation Sequencing Platform at the University of Bern according to the protocols and kits of Pacific Biosciences library preparation. The libraries were multiplexed on 1 × SMRTcell 8M using a Sequel IIe. After sequencing HiFi long reads, the demultiplexing was performed with lima, and the HiFi reads were quality controlled with FastQC and assembled using the version 11.0.0.146107 of the SMRTlink pb_microbial_analysis pipeline. The polishing, circularization, and origin rotation of the assembly are provided by the SMRTlink pipeline where required. The DNA modifications and methylated motifs were also identified by the SMRTlink pipeline. As expected for *Streptomyces* strains, the assemblies led to linear chromosomes of ∼8 Mbp with one or two plasmids from 80 to 162 kbp of which only one was found circularized. The completeness and contamination levels of the genomes were assessed using CheckM2 (Chklovski et al., 2023).

#### 2.2.3 Assembly annotation and analysis

The assemblies were compared using QUAST v. 4.6 (Mikheenko et al., 2016) and FastANI v. 1.33 (Jain et al., 2018), revealing that three strains belonged to the same species with an average nucleotide identity (ANI) > 95%. Close relatives were identified by Basic Local Alignment Search Tool searches in the National Center for Biotechnology Information GenBank public database: SS52 isolated from a plant in Vietnam (CP039123), WAC06273 isolated in a river in Nigeria (CP042278), and SGAir0924 isolated from Singapore air (CP027297). The genomes were annotated with Prokka (v. 1.13) (Seemann, 2014) and AntiSmash (v. 6.1.1) (Blin et al., 2021).

#### 2.2.4 ANI and tree

The heatmap displaying the ANI was calculated using ANIclustermap (v. 1.1.0) (Shimoyama, 2022) and fastANI (v. 1.33) (Jain et al., 2018). The core genome of the six genomes belonging to the B5 cluster was identified by ROARY (v. 3.11) (Page et al., 2015) and the phylogenetic tree of the core genomes was inferred by iqtree (v. 2.0.3) (Nguyen et al., 2015).

### 2.3 Assessment of the *Streptomyces* strains' activity against fungal and oomycete pathogens

To assess the effect of the three *Streptomyces* strains on the mycelial growth of different fungal and oomycete pathogens, three drops of 10 µL bacterial spore suspension at OD_600_ = 0.1 were pre‐inoculated on V8 plates at a 3 cm distance from the center of the plate and an equal distance between each other. The plates were incubated at 28°C for 48 h. Then, a 5‐mm‐diameter plug of the pathogen's mycelium was placed on the middle of the plate (control plates were inoculated only with a plug of each pathogen without bacteria). The plates were then sealed with Parafilm and incubated in the dark following the optimal growth temperature of each pathogen (see above). Pictures were taken after obtaining fully grown control plates and the area covered by mycelium growth was measured using the Image J software (Schneider et al., 2012). The growth inhibition percentage was calculated for each strain by comparing the mycelial growth percentage of the pathogens in the presence and absence of the bacteria. The equation employed was Ai = Ac − Ab − At, where Ai represents the inhibition area, Ac corresponds to the average of the area occupied by the pathogen in the control plates, Ab represents the area occupied by the bacteria in the test plate, and At indicates the area occupied by the pathogen in the test plate. The growth inhibition percentage was calculated using the formula: inhibition percentage = 100 × Ai/(Ac – Ab), with Ac – Ab representing the available area for the pathogen growth in the test plates (Bruisson et al., 2019). This experiment was replicated thrice with three technical replicates in each independent biological experiment. One‐way analysis of variance (ANOVA) test with Tukey's Honestly Significant Difference test (Tukey's HSD) was performed to determine whether there were statistically significant differences between the treatments.

### 2.4 Assessment of the *Streptomyces* strains' activity against bacterial pathogens

A cross‐streak method was used to assess the ability of the three *Streptomyces* strains to inhibit the growth of bacterial pathogens. First, each bacterial pathogen (*D. solani* and *P. carotovorum*) was inoculated by adding 2–3 colonies into a Falcon tube (Cellstar tubes; Greiner Bio‐One) containing 5 mL of LB. The tubes were incubated at 28°C and 180 rpm for 24 h and optical density was adjusted to OD_600_ = 0.1 with 0.9% NaCl (sodium chloride 99.5%; Acros Organics) before use. Two days before the start of the experiment, 10 µL of each *Streptomyces* spore suspension was streaked on V8 medium plates. The plates were pre‐incubated at 28°C for 48 h, after which three streaks of 10 µL of each bacterial pathogen solution (prepared as described above) were inoculated on the plate, perpendicularly to the pre‐inoculated *Streptomyces* streak. The streaks of pathogens were inoculated to reach the *Streptomyces* streak very closely, with a maximum distance of 3 mm. The plates were then incubated at room temperature in the dark. Pictures were taken after 3 days and Image J was used to analyze them. The growth percentage of the pathogens' colony was assessed by the formula: average (initial length of pathogen streak − final length of pathogen growth) × 100. An ANOVA with Tukey's HSD test was performed to determine whether there were statistically significant differences between the different treatments.

### 2.5 Ethyl acetate solid–liquid extraction of *Streptomyces* metabolites

The metabolites of each *Streptomyces* strain were extracted from solid plates using ethyl acetate. One milliliter of each strain's spore suspension (OD_600_ = 1) was fully spread on a V8 plate and incubated at 28°C for 1 week. A non‐inoculated V8 medium control plate was also incubated for the same time. Eight plates per strain or control were used for metabolite extraction. The one‐week‐old agar plates of each strain were cut into pieces of ∼0.5 cm^2^ for extraction. The pieces of agar were put into a glass Erlenmeyer in which 200 mL of ethyl acetate (Ethyl acetate; Thommen‐Furler AG) were added. The mixture was then sonicated for 5 min and stirred for 30 min. The stirring step was repeated twice adding 100 mL of ethyl acetate each. The three extracts of each sample were combined, filtered through a filter paper (Folded filters 4V; Cytiva) into a previously weighed glass round‐bottom flask and then evaporated using a rotary evaporator (Rotavapor RE120; Büchi Labortechnik) at 37°C. The resulting dried crude extract was weighed and then dissolved in 99.9% methanol (Thommen‐Furler AG) to obtain a final concentration of 10 µg/µL. Extracts were stored at −20°C until further use or analysis. The same protocol was applied to obtain methanolic extracts by replacing ethyl acetate with methanol.

### 2.6 Assessing the activity of the extracted metabolites on the growth of different pathogens

The activity of the *Streptomyces* extracted metabolites against the pathogens was examined using the following disk diffusion method: the organic extracts dissolved in methanol at the concentration of 10 µg/µL of each *Streptomyces* strain were spotted into Whatman paper disks of 6 mm diameter (Whatman AA disks; Cytiva) to obtain final amounts of 25, 50, and 100 µg on each disk. Methanol‐spotted and dried disks were used as controls. After placing the disks on V8‐medium plates, with a distance of 3 cm from the center and equidistant from each other, a plug of 5 mm diameter of each fungal or oomycete pathogen was placed in the middle of the plate. The bacterial pathogens, previously cultivated on V8 medium and incubated at 28°C for 24 h, and then resuspended to achieve an OD_600_ = 0.1 were swabbed on V8 plates before placing the disks on the plates. The plates were then incubated in the dark at 21°C for all pathogens except *P. ultimum*, *D. solani*, and *P. carotovorum*, which were incubated at room temperature. The experiment was performed once with three technical replicates.

### 2.7 Evaluating the inhibition activity of borrelidin against the different pathogens

Different concentrations of 97% pure borrelidin BIB3043 (Apollo Scientific) were tested against all pathogens under study to investigate this compound's contribution to the growth inhibition that was caused by the *Streptomyces* strain B5. Borrelidin was dissolved in methanol at 1 mg/mL and different volumes of the solution were added to 6 mm diameter Whatman paper disks to obtain final amounts of 1 ng, 10 ng, 50 ng 100 ng, 1 µg, and 2 µg per disk. Disk diffusion assays using the different borrelidin quantities against all the pathogens were conducted as described above for the organic extracts. The experiment included triplicates and the average inhibition percentage was calculated for each borrelidin amount. The half‐maximal inhibitory concentration (IC_50_) of borrelidin was obtained for each pathogen using a linear regression equation.

### 2.8 Liquid Chromatography–Mass Spectrometry (LC‐MS) analysis and data treatment

#### 2.8.1 LC‐MS parameters

Chromatographic separation was performed on a Vanquish Flex UPLC system (Thermo Fisher Scientific) interfaced with a Q‐Exactive Plus mass spectrometer (Thermo Fisher Scientific), using a heated electrospray ionization (HESI‐II) source. Thermo Scientific Xcalibur 3.1 software was used for instrument control. The LC conditions were as follows: column, Waters BEH C18 100 × 2.1 mm, 1.7 μm; mobile phase, (A) water with 0.1% formic acid; (B) acetonitrile with 0.1% formic acid; flow rate, 600 μL/min; injection volume, 2 μL; gradient, linear gradient of 2%−100% B over 10 min and isocratic at 100% B for 2 min. The optimized HESI‐II parameters were as follows: source voltage, 3.5 kV (pos); sheath gas flow rate (N_2_), 55 units; auxiliary gas flow rate, 15 units; spare gas flow rate, 3.0; capillary temperature, 350°C, S‐Lens RF Level, 50. The mass analyzer was calibrated using a mixture of caffeine, methionine–arginine–phenylalanine–alanine–acetate, sodium dodecyl sulfate, sodium taurocholate, and Ultramark 1621 in an acetonitrile/methanol/water solution containing 1% formic acid by direct injection. The data‐dependent tandem mass spectrometry (MS/MS) events were performed on the three most intense ions detected in full scan MS (Top3 experiment). The MS/MS isolation window width was 1 Da, and the stepped normalized collision energy was set to 15, 30, and 45 units. In data‐dependent MS/MS experiments, full scans were acquired at a resolution of 35,000 Full Width at Half Maximum (FWHM) (at *m/z* 200) and MS/MS scans at 17,500 FWHM both with an automatically determined maximum injection time. After being acquired in an MS/MS scan, parent ions were placed in a dynamic exclusion list for 3.0 s.

#### 2.8.2 Data processing and molecular networking

The MS data were converted from .RAW (Thermo) standard data format to .mzML format using the MSConvert software, part of the ProteoWizard package (Chambers et al., 2012). The converted files were treated using the MZmine software suite v. 2.53 (Pluskal et al., 2010). The parameters were adjusted as follows: the centroid mass detector was used for mass detection with the noise level set to 2.0E4 for the MS level set to 1, and to 0 for the MS level set to 2. The ADAP chromatogram builder was used and set to a minimum group size of scans of 5, minimum group intensity threshold of 2.0E4, minimum highest intensity of 2.0E4, and *m/z* tolerance of 12 ppm (Myers et al., 2017). For chromatogram deconvolution, the algorithm used was the wavelets (ADAP). The intensity window signal‐to‐noise (S/N) was used as an S/N estimator with a S/N ratio set at 15, a minimum feature height at 2.0E4, a coefficient area threshold at 80, a peak duration range from 0.02 to 1.00 min and the retention time (RT) wavelet range from 0.02 to 0.05 min. Corresponding MS2 was paired with the following parameters (0.025 Da and 0.15 min). Isotopes were detected using the isotope peaks grouper with a *m/z* tolerance of 8 ppm, an RT tolerance of 0.08 min (absolute), the maximum charge set at 4, and the lowest *m/z* was used as the representative isotope. Peak alignment was performed using the join aligner method (*m/z* tolerance at 12 ppm), absolute RT tolerance 0.08 min, weight for *m/z* at 30, and weight for RT at 30. The aligned feature list (73,046 features) was exported using the export to Global Natural Product Social Molecular Networking (GNPS) Feature‐Based Molecular Network (FBMN) module. The MZmine parameters used for the data treatment are available at https://doi.org/10.25345/C5N87391K.

To analyze the spectral diversity of the profile collection, a molecular network (MN) was created on the GNPS website (http://gnps.ucsd.edu) using the .mgf spectra file generated at the previous step (Wang et al., 2016). The precursor ion mass tolerance was set to 0.02 Da and an MS/MS fragment ion tolerance of 0.02 Da. A network was then created where edges were filtered to have a cosine score above 0.7 and more than six matched peaks. Further, edges between two nodes were kept in the network if and only if each of the nodes appeared in each other's respective top 10 most similar nodes. Finally, the maximum size of a spectral family was set to 100, and the lowest‐scoring edges were removed from molecular families until the molecular family size was below this threshold. The spectra in the network were then searched against GNPS spectral libraries. All matches kept between network spectra and library spectra were required to have a score above 0.7 and at least six matched peaks. The resulting MN is available online at https://gnps.ucsd.edu/ProteoSAFe/status.jsp?task=3aa60b6233c84abb9ceda7087b9cca78. The converted mass spectrometry data files, the corresponding metadata table, and the metabolite annotation results table, together with a Cytoscape file corresponding to the full MN annotated with the experimental and theoretical spectral matches are available under the MassIVE ID MSV000092173.

### 2.9 Metabolite annotation

#### 2.9.1 Experimental spectral libraries search

The full spectral data set corresponding to the extracts collection has been uploaded on the MassIVE repository https://doi.org/10.25345/C5N87391K, and a continuous identification workflow is automatically carried against the Global Natural Products Social Molecular Networking (GNPS) experimental spectral libraries: https://gnps.ucsd.edu/ProteoSAFe/result.jsp?task=af3dbe84bc4c4fe38789c835722733ff&view=advanced_view.

#### 2.9.2 Theoretical spectral libraries search

In addition to experimental spectral libraries search we have shown that spectral matching against theoretical spectral libraries of natural products was an efficient way to cover a much wider, yet relevant, spectral space (Allard et al., 2016). Furthermore, we showed that taking into account the taxonomical distance between the biological source of the candidate structure and the biological source of the annotated extracts greatly improved the overall quality of the annotation results (Rutz et al., 2019). Thus, in addition to the spectral search performed at the molecular networking step against publicly available spectral libraries (see previous section) a taxonomically informed metabolite annotation was performed. For this, we first established a large theoretical spectral database of natural products following a previously established metabolite annotation workflow. This spectral database and associated biological sources metadata were constructed using chemical structure and information compiled during the LOTUS Initiative's first project aiming to establish an open and evolutive resource compiling natural products and biological occurrences (Rutz, Bisson, et al., 2022; Rutz, Sorokina, et al., 2022). The theoretical spectral database is publicly available (Allard et al., 2022). The biological sources metadata are available online (Rutz, Bisson, et al., 2022; Rutz, Sorokina, et al., 2022). The taxonomically informed metabolite annotation was performed using the met_annot_enhancer scripts version v0.1: https://github.com/mandelbrot-project/met_annot_enhancer/releases/tag/v0.1. The parameters used for the taxonomically informed metabolite annotation process and the resulting tables can be found at https://doi.org/10.25345/C5N87391K. Sirius (Dührkop et al., 2019) (v.5.5.7) and CANOPUS (Djoumbou Feunang et al., 2016; Dührkop et al., 2021; Kim et al., 2021) were also employed to proceed to metabolite annotation and attribution of chemical classes to MSMS spectra.

## 3 RESULTS AND DISCUSSION

### 3.1 The three strains belong to the same *Streptomyces* species, yet their genomes carry distinct BGCs

**Figure 1:**
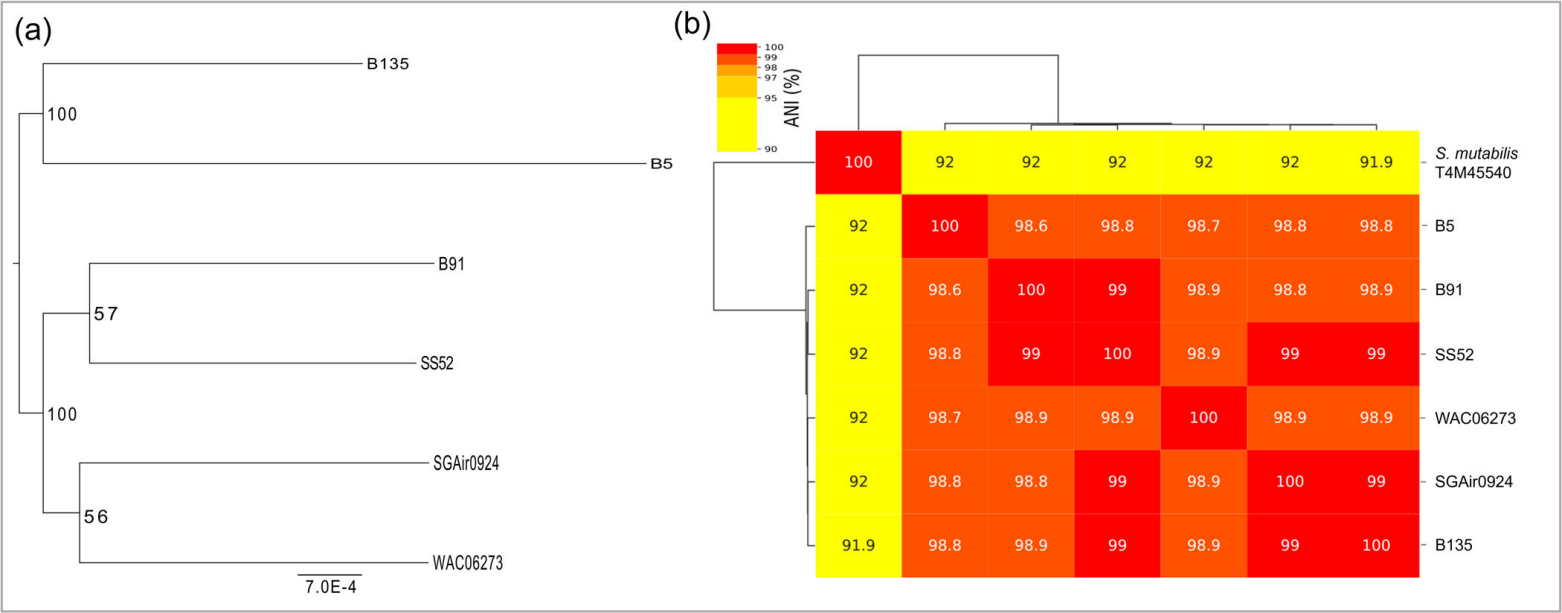
(a) Phylogenetic tree of *Streptomyces* strains B5, B91, and B135. The tree was constructed using ROARY and iqtree with bootstrap values expressed as a percentage of 1000 replicates. Genomes of the closely related strains SS52 (CP039123), WAC06273 (CP042278), and SGAir0924 (CP027297) were included in the analysis. (b) Heatmap showing the average nucleotide identity (ANI) generated with ANIclustermap and fastANI.

The three *Streptomyces* strains (B5, B91, and B135) demonstrated a close phylogenetic relationship based on 16S rRNA gene homology. Nevertheless, they showed varying degrees of inhibition against *P. infestans* mycelial growth. To examine the potential for specialized metabolite production and identify possible differences among the strains, the complete genome of each strain was sequenced. The assemblies resulted in a linear chromosome of approximately 7.87 Mbp and a linear plasmid of ∼81 kbp for *Streptomyces* B5, a linear chromosome of ∼7.99 Mbp, a linear plasmid of ∼87 kbp and a circular plasmid of ∼128 kbp for *Streptomyces* B91, and finally a linear chromosome of ∼7.99 Mbp, and a linear plasmid of ∼162 kbp for *Streptomyces* B135. The genome sequences of the three strains exhibited 100% completeness with less than 0.25% contamination. These genome sequences, along with their respective plasmids, have been deposited in the European Nucleotide Archive (PRJEB59914) under the accession numbers ERR11144646, ERR11144737, and ERR11145445 for B5, B91, and B135, respectively. Genome analysis of the three *Streptomyces* strains showed that they are not only close relatives, but that they belong to the same species with an ANI > 95%. This species has been previously reported in Vietnam (Nguyen et al., 2019), Nigeria (Culp et al., 2019), and Singapore (Gupta et al., 2019), and it has an ANI of 92% with *S. mutabilis* T4M45540, as shown in Figure 1.

Biosynthetic Gene Clusters (BGCs) were detected in each strain using antiSMASH v. 6.1.1 online software (Blin et al., 2021), and a summary of this analysis is presented in Table S1. The three strains shared 19 BGCs with the same percentage of genes showing similarity with the respective clusters reported in the MIBiG database. Among these 19 shared BGCs, some were previously reported to encode products conserved across the *Streptomyces* genus. These include ectoine, which prevents osmotic stress, geosmin, responsible for the characteristic odor of moist soil, hopene, which plays a role in prokaryotic membrane stability, spore pigments, as well as the siderophore desferrioxamine (Alam et al., 2022; Ward & Allenby, 2018). Additionally, the three genomes contained other BGCs encoding various antibiotics, such as albaflavenone (Zhao et al., 2008), candicidin (Chen et al., 2003), calcium dependant‐antibiotics (Hojati et al., 2002), and streptovaricin (Liu et al., 2017). Furthermore, the linear plasmids of the three strains carried the BGC encoding the antifungal aromatic polyketide fluostatins M‐Q (Jin et al., 2018) with a match percentage of 62% for both B5 and B135 and 69% for B91. Although it was anticipated that strains belonging to the same species would have high genomic similarity, some BGCs were not shared by all three genomes. The BGCs encoding lactonamycin and pristinin A3 were present in the genomes of B5 and B135 but not in B91. Coelichelin, granaticin, and versipelostatin were only detected in the genomes of B91 and B135, while no BGC was shared between the genomes of B5 and B91. Other BGCs were exclusively found in one of the strains: BGCs encoding alanylclavam and borrelidin were unique to B5, those encoding cacaoidin, hydroxystreptomycin, and methylenomycin A were only present in B91, and finally, those encoding 7-prenylisatin, isorenieratene, and streptothricin were unique to B135.

### 3.2 The three *Streptomyces* strains show different activity spectra on the different pathogens

**Figure 2:**
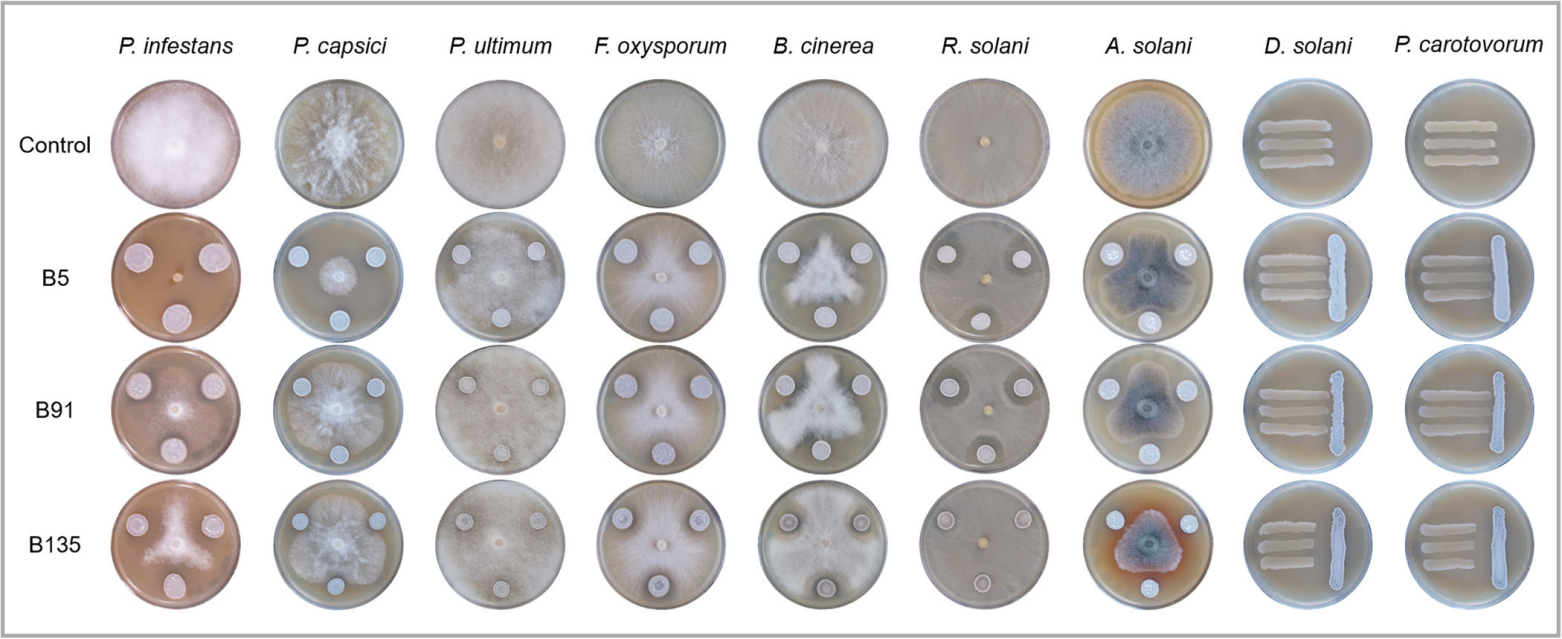
Representative plates showing the growth of different pathogens when co‐incubated with *Streptomyces* strains B5, B91, and B135. The represented pathogens are the oomycetes *Phytophthora infestans*, *Phytophthora capsici*, and *Pythium ultimum*, the fungi *Fusarium oxysporum*, *Botrytis cinerea*, *Rhizoctonia solani*, and *Alternaria solani*, and the bacteria *Dickeya solani* and *Pectobacterium carotovorum*.

The results of our activity assay on various pathogens (Table 1, Figure 2) first allowed us to confirm the previously observed differential activity of the three strains against *P. infestans* (Abdelrahman et al., 2022): B5 almost fully inhibited *P. infestans* (99%), while B135 showed an intermediate activity (61%) and B91 was relatively inactive (27%). A similar trend was observed for another oomycete pathogen, *P. capsici*, which was also most inhibited by B5 (82%), while it was less affected by the two other strains. In contrast, *P. ultimum* was not or only very marginally inhibited by any of the strains. The different inhibition observed against the three oomycetes might be at least partly due to their growth speed, with *P. ultimum* needing only 3 days to cover the plate, while it took *P. capsici* 5–6 days and *P. infestans* 10–14 days to do so. Therefore, *P. ultimum* might have grown faster than the time needed for *Streptomyces* metabolites to accumulate to sufficiently high amounts for mycelial growth inhibition to occur. Such a negative correlation between growth speed and sensitivity to bacterial metabolites has been observed in earlier studies dealing with volatile metabolites (Bailly & Weisskopf, 2017) and is expected to concern nonvolatile metabolites as well. It is worth noting that a radial inhibition phenotype was observed upon the co‐incubation of B5 and *P. infestans*. To verify whether this phenotype might be due to volatile‐mediated antibiosis, a split plate assay was conducted to investigate the contribution of volatiles to the observed activity. These results showed no volatile‐mediated activity of B5 against *P. infestans*, implying that the observed activity was indeed caused by diffusible compounds.

**Table 1:** Inhibitory activity of *Streptomyces* strains B5, B91, and B135 against multiple oomycete, fungal, and bacterial plant pathogens presented as inhibition percentages compared to the control (0% inhibition).

	B5	B91	B135
*Phytophthora infestans*	**99 (d)**	**27 (b)**	**61 (c)**
*Phytophthora capsici*	**82 (c)**	14 (ab)	**20 (b)**
*Pythium ultimum*	**9 (b)**	0 (a)	1 (a)
*Fusarium oxysporum*	16 (ab)	**22 (b)**	10 (ab)
*Botrytis cinerea*	**55 (c)**	**48 (c)**	**23 (b)**
*Rhizoctonia solani*	**27 (b)**	**24 (b)**	1 (a)
*Alternaria solani*	**49 (b)**	**64 (c)**	**68 (c)**
*Dickeya solani*	0 (a)	0 (a)	**24 (b)**
*Pectobacterium carotovorum*	0 (a)	0 (a)	**24 (b)**

*Note*: The assay was conducted independently three times with four technical replicates each. Letters indicate significant differences between treatments according to an analysis of variance followed by Tukey's Honestly Significant Difference test (*p* < 0.05). The percentages in bold are significantly different from the control.

Compared with the oomycetes, a different pattern of activity was observed when considering the effects of the three *Streptomyces* strains on true fungi: there, B5 and B91 displayed a similar level of inhibition of the Ascomycete *B. cinerea* (55% for B5, 48% for B91) and the Basidiomycete *R. solani* (27% for B5, 24% for B91), while B135 was the least active strain in both cases. Here too, the faster‐growing fungus (*R. solani*) was less strongly inhibited than the slower‐growing one (*B. cinerea*). Two additional Ascomycete pathogens were tested: *F. oxysporum*, which was only significantly affected by B91 (22%) but was still kept at some distance by the two other strains (Figure 2), and *A. solani*, which was strongly inhibited by the strains B135 (68%) and B91 (64%), while an intermediate inhibition was observed in its interaction with B5 (49%). Interestingly, the otherwise relatively inactive strain B135 not only strongly reduced the progression of *A. solani* mycelial growth, but also induced the production of melanin pigments by the fungus, which could be a sign of stress. Indeed, the production of melanin pigments in fungi has been reported to increase their protection against environmental stresses such as ultraviolet light, as well as to increase their virulence (Campana et al., 2022; Eisenman & Casadevall, 2012; Geib et al., 2016). *A. solani* was already known to constitutively produce melanin pigments and our results suggest that the fungus might increase their production, and/or release them into the medium when in confrontation with the *Streptomyces* strain B135 (Parvin et al., 2021). Furthermore, B135 also strongly inhibited the growth of the two bacterial pathogens, which were not at all affected by the two other *Streptomyces* strains (Figure 2). This suggests the occurrence of a unique antibiotic inhibiting the growth of Gram‐negative bacteria in B135. Interestingly, among the three unique BGCs encoded in B135, two encoded products are reported to exhibit antibacterial activity. One of these is 7‐prenylisatin, a prenylated isatin compound previously reported in *Streptomyces* sp. MBT28, which demonstrated antibacterial activity against the Gram‐positive *Bacillus subtilis* (Wu et al., 2015). The other is streptothricin, which comprises a streptolidine base and is known for its antibacterial properties (Ashraf et al., 2021; Ji et al., 2009).

To summarize the activity demonstrated by the three strains, B5 showed the highest inhibition of oomycetes, B5 and B91 the highest inhibition of fungi, and B135 the highest inhibition of bacteria. The differential activity of the three strains on the different types of pathogens suggests the production of different molecules involved in the respective inhibitions in a specific manner, rather than the production of a broad range toxins, which would affect all organisms similarly. This differential activity pattern also allows a specific search for compounds uniquely present in B5 or B135 to explain their respective anti‐oomycete and antibacterial/melanin‐inducing activity, or for compounds shared by B5 and B91 to explain their antifungal activity.

### 3.3 Metabolites extracted from *Streptomyces* B5 had similar anti‐oomycete activity as the strain itself

**Figure A1:**
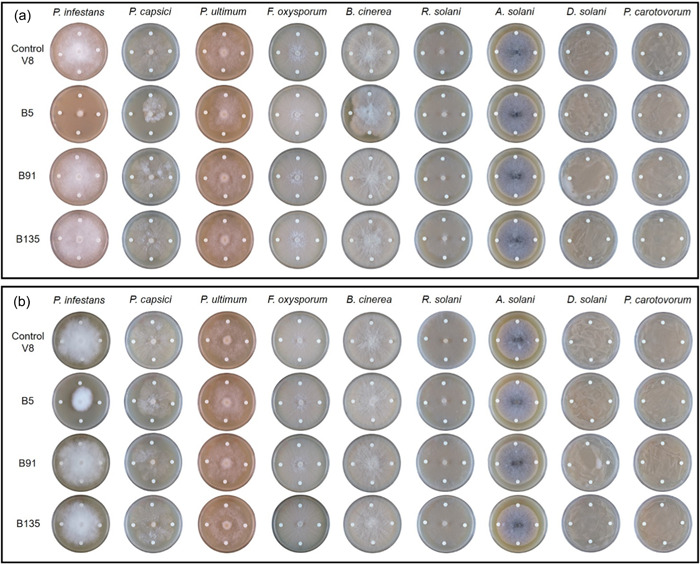
Representative plates showing the pathogen growth in the presence or absence of the organic extracts (with (a) ethyl acetate and (b) methanol) of *Streptomyces* strains B5, B91, and B135 against multiple plant pathogens. Several extract amounts resuspended in methanol were spotted and dried on the disks; 100 (left side), 50 (lower side), and 25 µg (right side), while methanol was used as blank (upper side). The experiment was performed once with two technical replicates.

**Figure A2:**
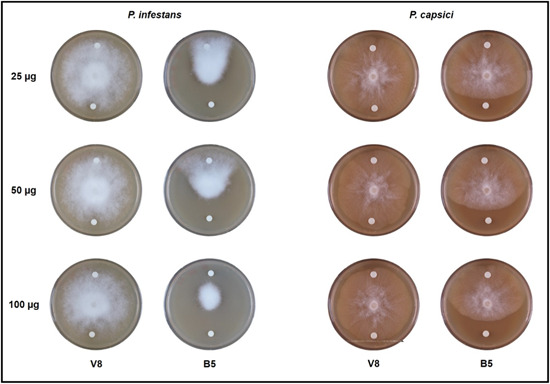
Representative plates showing the growth of two *Phytophthora* species in the presence or absence of the metabolites produced by *Streptomyces* strain B5 and extracted with ethyl acetate. The upper disks are methanol blanks, and the lower disks are the different amounts tested.

As a step toward identifying the bioactive compounds produced by each strain, the activity of the ethyl acetate and methanolic extracts of the strains was evaluated using the disk diffusion method. Notably, the metabolites extracted from the *Streptomyces* strain B5 exhibited significant activity against the three oomycetes, against *B. cinerea* as well as—to a lower extent—against *A. solani*, while no activity of the extracts was observed against the other pathogens that were inhibited by the strain itself. The ethyl acetate extract of B5 displayed higher efficacy in inhibiting the growth of the pathogens compared to the methanolic extract of the same strain (Figure A1). This observation suggests that ethyl acetate extracted most of the active compounds responsible for B5's activity, while the more polar solvent methanol was less suitable for extracting them. Furthermore, the extracted metabolites of B5 exhibited the same activity pattern on oomycetes as the strain itself, with *P. infestans* being the most inhibited, followed by *P. capsici*, and finally by *P. ultimum* (Figures A1 and A2).

In contrast, despite the observed inhibitory potential of both B91 and B135 against various pathogens, their extracts were completely inactive against all pathogens examined (Figure A1). This suggests that the active molecule(s) might not have been successfully extracted using our protocol, for example, because of their protein nature. Another possible explanation for the lack of activity could be that the active metabolites responsible for the activity of these strains are not constitutively produced, but rather induced upon interaction with the respective pathogen. Previous studies have demonstrated that specialized metabolite production in microorganisms can be induced through interactions with volatile or nonvolatile compounds (Bruisson et al., 2023; Weisskopf et al., 2021). Furthermore, co‐culturing *Streptomyces* with other bacteria and fungi has been employed as an approach to stimulate silent or cryptic metabolic pathways by activating various BGCs present in the *Streptomyces* genomes that remain unexpressed under laboratory conditions (Nicault et al., 2021; Onaka, 2017).

### 3.4 Metabolic variations among the three strains shed light on their differential bioactivities

**Figure 3:**
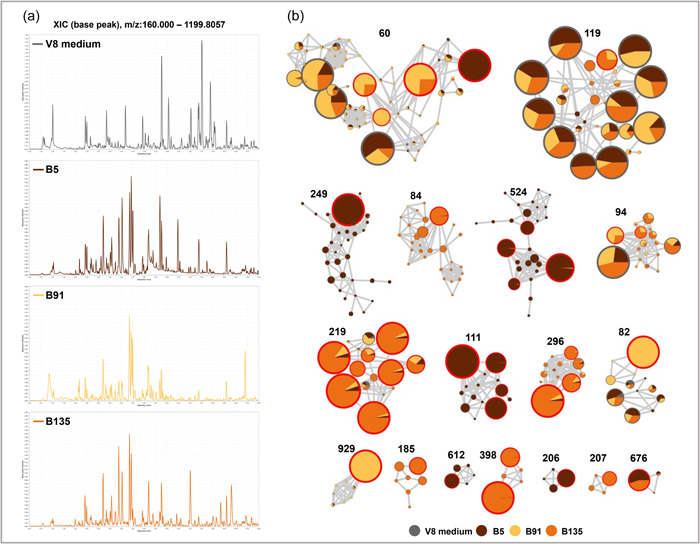
(a) Total ion chromatograms (positive mode) of EtOAc extracts (5 µg/µL) of *Streptomyces* strains B5, B135, B91, and V8 medium. (b) Selected clusters include the most intense features observed in the feature‐based molecular network of EtOAc extracts of B5, B135, B91, and V8 medium. The cluster ID is indicated above each cluster. The color of each node represents the corresponding sample, while the size of the node indicates the summed precursor intensity of the feature. The pie chart within each node represents the relative abundance of the corresponding feature in the different samples. Nodes with red borders correspond to the most intense features that were either unique to or highly abundant in one of the extracts or shared between the two of them. The putative annotation of the selected nodes is provided in Table S2.

Ultrahigh performance liquid chromatography coupled with high‐resolution tandem mass spectrometry analysis was conducted on the ethyl acetate extracts obtained from the three *Streptomyces* strains, together with the V8 medium extract used as a negative control. The total ion chromatograms of the strains displayed relative similarity with many shared peaks as depicted in Figure 3a. To visualize differences among the strains' chemical profiles more effectively, the acquired data were utilized to construct a Feature‐Based Molecular Network (FBMN) (Nothias et al., 2020) aimed at unveiling the variations in the metabolomic profiles among the three strains, which could potentially contribute to the observed variations in their respective activities. The data set comprised a total of 19,993 spectra, which were organized into 7485 nodes. Among these nodes, 5353 were grouped into 1555 distinct clusters, while the remaining 2132 nodes were classified as singletons, indicating a lack of spectral similarity with other nodes under the experimental conditions used to generate the FBMN (cosine threshold of 0.7). Out of the 1555 clusters, our attention was directed toward 17 clusters that contained the most intense features, exhibiting a summed precursor intensity of ≥1.02E + 08. These clusters consisted of features that were either exclusive or highly abundant in one of the strains' extracts or shared between the extracts of two strains but absent in the third. Importantly, these 17 clusters did not include features with high abundance in the control V8 medium extract (Figure 3b). By applying the aforementioned selection criteria, 37 features were found (indicated by red borders in Figure 3b), 12 of which exhibited uniqueness or high abundance in B5, 3 in B91, and 16 in B135. One feature was shared exclusively between B5 and B135 extracts, while 5 features were shared exclusively between B91 and B135 extracts. However, no feature within the selected clusters was found to be shared between B5 and B91 extracts, confirming the antiSMASH analysis results at the genome level.

Among the selected features, 36 were putatively annotated using (i) taxonomically informed spectral matching against a theoretical spectral database of natural products (built from the LOTUS resource) (Allard et al., 2016; Rutz, Bisson, et al., 2022; Rutz et al., 2019; Rutz, Sorokina, et al., 2022) and (ii) metabolite and chemical class annotation using the SIRIUS pipeline (Djoumbou Feunang et al., 2016; Dührkop et al., 2019, 2021; Kim et al., 2021).

The resulting annotations can be found in Table S2. Among the putatively annotated metabolites, two notable compounds were exclusively present in the extract of B5: the esterase inhibitor esterastin (cluster 60, feature 29050), and the angiogenesis inhibitor borrelidin (cluster 111, feature 29069). Both compounds have been previously reported in *Streptomyces* sp. (Umezawa et al., 1978; Wakabayashi et al., 1997). Another compound, terrein (cluster 82, feature 49923), was exclusively found in B91's extract and is known for its antifungal, anticancer, moderate antibacterial, and antioxidant activities (Goutam et al., 2017). Terrein is primarily produced by various *Aspergillus* sp. (Hill et al., 1981; Nagia et al., 2012), as well as *Penicillium* sp. (Park et al., 2004) and *Streptomyces* sp. (Ma et al., 2018). Furthermore, the predicted molecular formula and structure of feature 31099 (cluster 398), which exhibited high abundance in the extract of B135, were found to be similar to those of the known antibacterial compound 7-prenylisatin, previously identified exclusively in the genome of B135. Taken together, this suggests that feature 31099 is likely to be 7‐prenylisatin.

Interestingly, upon examining the metabolites whose BGC were encoded in the genomes of the three strains, only borrelidin, specific to B5's genome, and 7‐prenylisatin, specific to B135's genome, were detected in the extracts of the respective strains. This observation highlights that most of the metabolites predicted to be produced by the strains based on their genome analysis were not detected in their extracts under our experimental conditions. This could suggest, on the one hand, that antiSMASH predictions are more accurate for BGCs displaying a high percentage of matching genes, indicating high conservation of the cluster within the respective genome, but on the other hand, this discrepancy between genome‐based predictions and metabolite detection could be also due to the influence of environmental factors or specific growth conditions on metabolite production. This finding provides a potential explanation for the lack of observed activity when assessing the effect of B91 and B135 extracts on the growth of the pathogens (Figure A1), as the metabolites responsible for the strains' activity might not be constitutively produced but only as a result of the interaction between the two organisms (*Streptomyces* sp. vs. pathogen) and might therefore be absent in the extracts harvested from *Streptomyces* pure cultures.

### 3.5 Borrelidin is responsible for B5's activity

Upon overlaying the total ion chromatograms of the ethyl acetate extracts from the three *Streptomyces* strains, we observed a distinctive peak with high intensity in the chromatogram of B5. This peak eluted at 6.47 min with *m/z* of 472.31 (Figure 3a). Additionally, another unique peak was detected in the chromatogram of B5, which eluted at 6.94 min with *m/z* of 490.32. Through data analysis, we identified two corresponding features: feature 29041, corresponding to the peak eluting at 6.47 min, and feature 29069, corresponding to the peak eluting at 6.94. Interestingly, when visualizing the constructed FBMN, these two features were grouped in cluster 111, which comprised a set of features unique to B5's extract. Therefore, these features are expected to be structurally related due to their spectral similarity. The putative annotation of feature 29069 indicated the molecular formula C_28_H_43_NO_6_ and the annotation (via SIRIUS) as borrelidin [M + H]^+^. Notably, borrelidin was indicated to be encoded in the genome of B5 as a unique BGC that was absent from the genomes of the two other strains, hence strengthening this putative identification. The molecular formula of feature 29041 was tentatively annotated as C_28_H_41_NO_5_ and further analysis of this feature and its associated spectra suggested that it could correspond to a dehydrated (‐H_2_O) analogue of borrelidin.

Borrelidin is a polyketide macrolide that was initially discovered in a strain of *Streptomyces rochei* isolated from a soil sample and found to exhibit anti‐*Borrelia* activity (Berger et al., 1949). Subsequently, borrelidin was identified in other *Streptomyces* species and shown to possess various biological activities. For instance, it was produced by *Streptomyces* sp. strain C2989 and exhibited antiviral activity (Lumb et al., 1965). It was also found in *S. albovinaceous*, where it demonstrated antibacterial activity against *Treponema hyodysenteriae* and was hence named treponemycin (Singh et al., 1985). Later on, treponemycin was found to be identical to borrelidin (Maehr & Evans, 1987). Borrelidin was additionally described as an angiogenesis inhibitor (Wakabayashi et al., 1997) and its BGC was cloned from *S. parvulus* strain Tü4055 and analyzed by Olano et al. (2004). It has also shown antimalarial activity as reported by Otoguro et al. (2003) and Tamaki et al. (2022). Furthermore, borrelidin has been isolated from the soil isolate *S. californicus* (Srinivasan et al., 2008), and the endophytic isolate *S. heilongjiangensis*, which was obtained from the root surface of soybean (Liu et al., 2013). Shiang et al. (2001) were the first to describe the anti‐oomycete activity of borrelidin against several species, including *Pythium aphanidermatum*, *Pythium splendens*, *Pythium sylvaticum*, *P. ultimum*, and *P. capsici*. Borrelidin was also found to exhibit strong antioomycete activity against eight races of *Phytophthora sojae* surpassing the activity of the commercial fungicide metalaxyl used for *P. sojae* control (Liu et al., 2012). Gao et al. (2012) suggested that the anti‐oomycete activity of borrelidin against *P. sojae* is mediated by the inhibition of threonyl‐tRNA synthetase (ThrRS) through the formation of the ThrRS−borrelidin complex. Additionally, borrelidin was identified as the antioomycete compound produced by *Streptomyces plicatus* strain B4‐7, which inhibited *P. capsici* (Chen et al., 2016).

**Figure A3:**
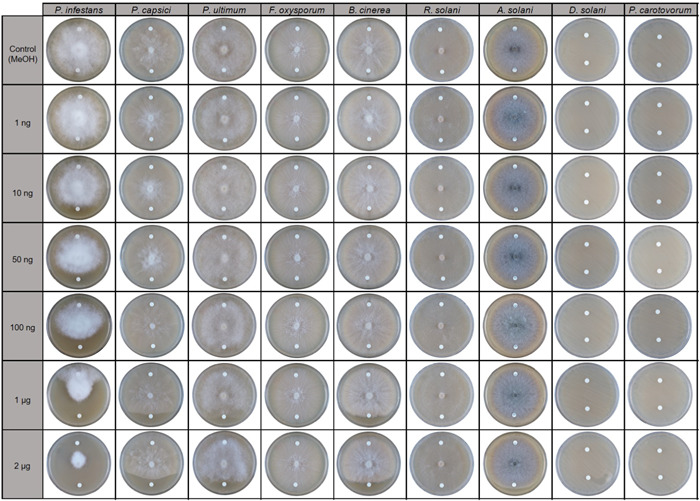
Representative plates showing the growth of different pathogens in the presence or absence of different amounts of borrelidin. The upper disks are methanol blanks, and the lower disks are the different concentrations tested. The experiment was performed once with three technical replicates.

Considering the reported activities of borrelidin and the putative annotation of borrelidin in B5 extract, which exhibited strong activity against the oomycetes used in this study as well as against *B. cinerea* and *A. solani*, a pure borrelidin standard was obtained to evaluate its activity against all the pathogens investigated and to confirm the putative annotation. Remarkably, the borrelidin standard exhibited the same activity profile as the ethyl acetate extract of B5. It displayed strong inhibitory activity against *P. infestans* followed by *P*. capsici, *B. cinerea*, and *P. ultimum*, and a slight activity against *A. solani* with IC_50_ values of 0.99, 4.02, 5.69, 9.48, and 41.13 µg, respectively (Figure A3). The inhibitory activity of borrelidin increased with higher concentrations, indicating a dose‐dependent effect, as previously described for its activity against *P. sojae* (Liu et al., 2012).

**Figure 4:**
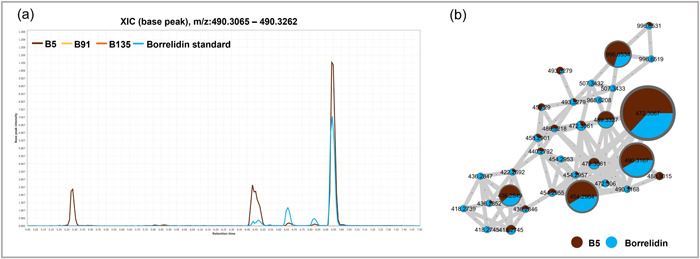
(a) Observation of the extracted ion chromatograms (for molecular formula of borrelidin C_28_H_43_NO_6_) indicates the presence of borrelidin in B5's EtOAc extract (5 µg/µL) and its absence from the extracts of B91 and B135 when compared to the pure standard (1 µg/µL). Note that the extracted ion chromatogram corresponding to the pure standard indicates the presence of multiple isomers. (b) Cluster corresponding to the borrelidin compound observed in the FBMN of the ethyl acetate extract of *Streptomyces* sp. strain B5 and of the borrelidin standard. The node size indicates its summed precursor intensity. FBMN, Feature‐Based Molecular Network; XIC, extracted ion chromatograms.

LC‐MS analysis of the pure borrelidin standard, compared to the putative borrelidin in B5, confirmed our initial annotation. The standard exhibited the same distinctive peaks as B5's extract when the extracted ion chromatograms (XIC) were overlaid using the borrelidin formula [M + H]^+^ for the three *Streptomyces* extracts (5 µg/µL) and borrelidin standard (1 µg/µL) as shown in Figure 4a. Furthermore, the features of the borrelidin standard grouped with B5's unique features in the same cluster when constructing a new FBMN of the samples and the standard (Figure 4b). This indicates that the bioactivity of the ethyl acetate extract of the *Streptomyces* strain B5 can be mostly attributed to borrelidin. It is important to note that the purchased borrelidin standard contains a mixture of borrelidin isomers since four different peaks with the same mass were observed in the LC‐MS profile of the standard. Moreover, an additional peak (eluting at 5.29 min with *m/z* 490.32) was observed in B5's XIC, which was extracted using the borrelidin molecular formula. However, this peak was not detected in the borrelidin standard XIC (Figure 4a). Furthermore, the feature corresponding to this peak did not cluster with the other borrelidin features and appeared as a singleton in the FBMN. These findings suggest that B5 produces an isomer of borrelidin that is not present in the commercially purchased standard. Nevertheless, rough quantification of borrelidin in B5 extract was performed by constructing an LC‐MS standard curve using different concentrations of the borrelidin standard, revealing that borrelidin accounts for approximately one‐tenth of the extracted metabolites from B5.

Although borrelidin has been previously described as having anti‐*Phytophthora* spp. and anti‐*Pythium* spp. activity, this is the first study, to the best of our knowledge, that demonstrates its anti‐*P. infestans* properties and its antifungal activity against some of the true fungi such as *B. cinerea*. Given the high relevance of *P. infestans* as a disease‐causing agent in both potato and tomato, these findings highlight borrelidin as an important target and desirable trait in the search for biocontrol strains with the potential for the sustainable control of this destructive pathogen. Furthermore, this study revealed that morphologically and genetically similar *Streptomyces* strains can exhibit substantial variations in their inhibitory potential against different pathogens and produce a distinct set of metabolites. This finding emphasizes the effectiveness of comparative omics approaches on phylogenetically closely related strains of differing activities to identify new or previously known bioactive compounds that could be utilized for plant disease control. Further investigations are warranted to evaluate the effects of borrelidin against phytopathogens *in planta*. Moreover, and going beyond the strong anti‐*Phytophthora* activity of B5 this study focused on, our work provides a series of candidate molecules to be further investigated to identify the metabolites that may account for the other biological activities demonstrated by the three *Streptomyces* strains examined in this study.

## AUTHOR CONTRIBUTIONS

**Alisson Gillon**: Formal analysis (supporting); investigation (equal); methodology (supporting); validation (supporting); visualization (equal); writing—original draft (supporting); writing—review and editing (supporting). **Ola Abdelrahman**: Conceptualization (equal); data curation (lead); formal analysis (equal); investigation (lead); methodology (equal); supervision (supporting); validation (equal); visualization (equal); writing—original draft (lead); writing—review and editing (equal). **Eliane Abou‐Mansour**: Conceptualization (supporting); methodology (supporting); resources (equal); supervision (supporting); writing—review and editing (supporting). **Floriane L'Haridon**: Project administration (supporting); resources (equal); supervision (supporting); writing—review and editing (supporting). **Laurent Falquet**: Conceptualization (supporting); data curation (equal); methodology (equal); resources (equal); software (equal); supervision (supporting); writing—review and editing (supporting). **Pierre‐Marie Allard**: Conceptualization (supporting); data curation (equal); methodology (equal); software (lead); supervision (equal); visualization (supporting); writing—original draft (equal); writing—review and editing (supporting). **Laure Weisskopf**: Conceptualization (equal); funding acquisition (lead); project administration (lead); supervision (lead); writing—original draft (equal); writing—review and editing (lead).

## CONFLICT OF INTEREST STATEMENT

None declared.

## ETHICS STATEMENT

None required.

## Supporting information

Supporting information.

Supporting information.

## Data Availability

All data required for this study are included in this paper, its supplementary materials, and through the provided hyperlinks. The full spectral data set corresponding to the extract collection has been uploaded to the MassIVE repository https://doi.org/10.25345/C5N87391K. The genome sequences of the three *Streptomyces* strains can be accessed in the European Nucleotide Archive under the project accession number PRJEB59914, with individual accession numbers as follows: ERR11144646 for B5, ERR11144737 for B91, and ERR11145445 for B135: https://www.ebi.ac.uk/ena/browser/view/PRJEB59914.

## 1

See Figures A1, A2, A3.
